# Compressed Symmetric Nested Arrays and Their Application for Direction-of-Arrival Estimation of Near-Field Sources

**DOI:** 10.3390/s16111939

**Published:** 2016-11-17

**Authors:** Shuang Li, Dongfeng Xie

**Affiliations:** 1 School of Electrical and Electronic Engineering, Chongqing University of Technology, Chongqing 400054, China; 2 Shanghai Huawei Technologies Co., Ltd., Shanghai 200120, China; dfhsie@126.com

**Keywords:** sensor array signal processing, direction-of-arrival estimation, near-field, compressed symmetric nested array, fourth-order cumulants

## Abstract

In this paper, a new sensor array geometry, called a compressed symmetric nested array (CSNA), is designed to increase the degrees of freedom in the near field. As its name suggests, a CSNA is constructed by getting rid of some elements from two identical nested arrays. The closed form expressions are also presented for the sensor locations and the largest degrees of freedom obtainable as a function of the total number of sensors. Furthermore, a novel DOA estimation method is proposed by utilizing the CSNA in the near field. By employing this new array geometry, our method can identify more sources than sensors. Compared with other existing methods, the proposed method achieves higher resolution because of increased array aperture. Simulation results are demonstrated to verify the effectiveness of the proposed method.

## 1. Introduction

As one of the most important problems in sensor array signal processing, direction-of-arrival (DOA) estimation plays an important role in many applications such as radar, sonar, communication system, wireless sensor networks and some other fields. Therefore, it has drawn many researchers’ attention and plenty of methods [1] have been proposed to deal with this issue in the past few years, such as MUSIC [2], ESPRIT [3], etc. Most of these methods made the assumption that the sources are located relatively far from the array so that the waves emitted by the sources can be considered plane waves. However, when the sources are located close to the array, the wavefront should be characterized by both the DOA and range. Thus, existing methods based on the far-field assumption are not applicable to this situation.

In the past decades, numerous DOA estimation methods were presented in the near field, such as the two-dimensional MUSIC method [4], maximum likelihood method [4,5], the weighted linear prediction method [6] and higher-order-based methods [7,8,9,10,11]. However, these methods either require multidimensional search or suffer poor resolution from heavy aperture loss. Recently, some DOA estimation methods [12,13,14] based on sparse signal recovery have been developed for near-field sources, exhibiting several advantages compared to other methods. Nevertheless, these sparse-signal-recovery-based methods greatly rely on properly choosing the regularization parameter to reconstruct the sparse signal. Once the regularization parameter is improperly selected, the performance of the sparse-signal-recovery-based methods is greatly influenced. Unfortunately, there is no good way to choose the regularization parameter appropriately and it is still an open issue.

Besides, all the above DOA estimation methods for near-field sources are only applicable to the case of uniform linear arrays (ULA). The number of sources that can be identified with an *N*-element ULA using these methods is less than or equal to N−1. Nevertheless, there are several ways to implement underdetermined DOA estimation in the far-field [15,16,17,18,19,20,21,22,23,24]. Minimum redundancy array (MRA) [15] is the earliest array structure to conduct the underdetermined issue. However, there is no closed form for the array geometry and achievable degrees of freedom for a given array with N elements. Through fourth order cumulants, Dogan and Mendel [18] showed that one can extend the array aperture for an arbitrary array except a doublet is required in a physical array. To increase both the resolution and the number of sources to be detected, a novel underdetermined method named 2*q*-MUSIC [16] was proposed by Chevalier et al. by employing 2qth order cumulants. However, these higher-order-cumulants-based methods involve intensive computation. Though the Khatri-Rao product and only use of covariance, a method named KR-MUSIC was presented by Ma et al. [19], and using this approach 2N−2 quasi-stationary sources can be processed with an *N*-element ULA. Through the use of manifold separation technique, Cao et al. [20] extended KR-MUSIC to uniform circular array. However, the two methods [19,20] fail to process stationary sources. Based on the theory of sparse signal recovery, He et al. [21] proposed an underdetermined DOA estimation method for wideband signals. Vaidyanathan and his team [17,22,23,24] have presented some new array structures to conduct underdetermined DOA estimation. Nested array [17] is one of the best-designed array structures and is capable of significantly increasing the degrees of freedom (DOF), i.e., $O(N^{2})$ sources can be identified by exploiting a nested array with N sensors. Some other non-uniform array geometries include multiple level nested array [22], co-prime array [23] and super nested array [24]. By implementing DOA estimation on the virtual difference co-array of these non-uniform linear arrays, one can process more sources than sensors easily.

So far as we know, no existing methods can detect more sources than the number of physical sensors in the near-field. To bridge this gap, a novel array geometry, called a compressed symmetric nested array (CSNA), is proposed in this paper for the first time. A new underdetermined DOA estimation approach is also presented through the use of fourth-order cumulants in the near-field. By exploiting CSNA, the proposed DOA estimation method can process more sources than the number of sensors, i.e., as many as $N_{2}(N_{1}+1)-1$ sources can be detected using only $N=N_{1}+2N_{2}-1$ sensors in the near-field. Moreover, the proposed method can achieve higher resolution compared with other methods.

This paper is organized as follows. The data model and one existing method are presented in Section 2. Section 3 describes the details of the design of CSNA and its application for DOA estimation in near-field case. Several numerical experiments are given in Section 4. Section 5 concludes this paper.

In the rest of this paper, the superscripts T, * and H denote the transpose, the conjugation without transpose and conjugate transpose, respectively.

## 2. Data Model and One Existing Method

### 2.1. Model of DOA Estimation

**Figure 1:**
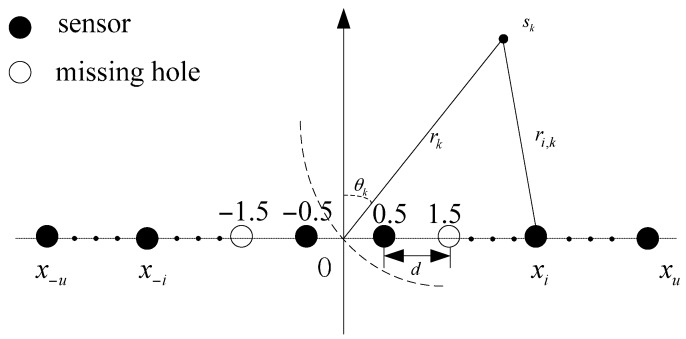
Non-uniform linear array configuration (The number of sensors is assumed to be even).

Consider a near-field scenario in which K narrowband sources impinge on a symmetric linear array with N=2u sensors showing Figure 1 (The number of sensor is assumed to be even here, however, our method also works in odd case). The sensors are assumed to be located at the underlying grid of a minimum spacing d. Let $x=\{x_{-u},\cdots ,x_{-2},x_{-1},x_{1},x_{2},\cdots ,x_{u}\}d$ be the positions vector of array sensors. The signal received by the ith sensor can be expressed as Equation (1)

(1) $$y_{i}(t)=\sum_{k=1}^{K}s_{k}(t)\exp(j\frac{2\pi }{\lambda }(r_{ik}-r_{k}))+n_{i}(t),\;t=1,2,\cdots ,T;\;i=-u,\cdots -1,1,\cdots ,u$$

where

(2) $$r_{ik}=\sqrt{r_{k}^{2}+x_{i}^{2}d^{2}-2x_{i}dr_{k}\sin\theta_{k}}$$

denotes the distance between the kth source and the ith sensor, $r_{k}$ represents the range from the kth source to the phase reference, $s_{k}(t)$ is the signal radiated from the kth source, λ denotes the wavelength, $n_{i}(t)$ is the received noise by the ith sensor and T stands for the number of snapshots.

Using the second-order Taylor expansion of Equation (2), we can approximate $\tau_{ik}≜\frac{2\pi }{\lambda }(r_{ik}-r_{k})$ as

(3) $$\begin{array}{ll} \tau_{ik} & ≜\frac{2\pi }{\lambda }(r_{ik}-r_{k})\\ & \approx -x_{i}\frac{2\pi d}{\lambda }\sin\theta_{k}+x_{i}^{2}\frac{\pi d^{2}}{\lambda r_{k}}\cos^{2}\theta_{k}\\ & =x_{i}\omega_{k}+x_{i}^{2}\varphi_{k} \end{array}$$

where

(4) $$\omega_{k}=-\frac{2\pi d}{\lambda }\sin\theta_{k}$$

and

(5) $$\varphi_{k}=\frac{\pi d^{2}}{\lambda r_{k}}\cos^{2}\theta_{k}$$

Substituting Equation (3) into Equation (1), we have

(6) $$y_{i}(t)=\sum_{k=1}^{K}s_{k}(t)e^{j(x_{i}\omega_{k}+x_{i}^{2}\varphi_{k})}+n_{i}(t)$$

Stacking the measurements of all the sensors in a vector form, we get

(7) y(t)=As(t)+n(t), t=1,2,⋯,T

where $y(t)={\left[y_{-u}(t),y_{-u+1}(t),\cdots ,y_{u}(t)\right]}^{T}$ is the observed signal vector, $A=[a(r_{1},\theta_{1}),a(r_{2},\theta_{2}),\cdots ,a(r_{k},\theta_{k})]$ denotes the array manifold, $a(r_{k},\theta_{k})={\left[e^{j\tau_{-u,k}},e^{j\tau_{-(u-1),k}},\cdots ,e^{j\tau_{u,k}}\right]}^{T}$ is the so-called steering vector, $s(t)={\left[s_{-u}(t),s_{-u+1}(t),\cdots ,s_{u-1}(t),s_{u}(t)\right]}^{T}$ and $n(t)={\left[n_{-u}(t),n_{-u+1}(t),\cdots ,n_{u-1}(t),n_{u}(t)\right]}^{T}$ are the source and noise vectors, respectively.

To make the following derivation simple, some assumptions are made as follows:

(A1) The sources are non-Gaussian, and mutually uncorrelated.
(A2) The noise is additive Gaussian one, either white or coloured, and independent of the sources.
(A3) The array is a non-uniform linear array with underlying grid $d\leq \frac{\lambda }{4}$ to avoid manifold ambiguity.ss

In this paper, we focus on solving the following DOA estimation problem: Given the received signals {y(t),t=1,2,⋯,T}, find the azimuth $\theta ={\left[\theta_{1},\theta_{2},\cdots ,\theta_{K}\right]}^{T}$.

### 2.2. Vitual Array Model by Exploiting Fourth Order Cumulants

The fourth order cumulant of the measured signals is defined as [18]

(8) $$\begin{array}{l} cum(y_{m}(t),y_{n}^{*}(t),y_{p}(t),y_{q}^{*}(t))=E\{y_{m}(t)y_{n}^{*}(t)y_{p}(t)y_{q}^{*}(t)\}\\ -E\{y_{m}(t)y_{n}^{*}(t)\}E\{y_{p}(t)y_{q}^{*}(t)\}-E\{y_{m}(t)y_{p}(t)\}E\{y_{n}^{*}(t)y_{q}^{*}(t)\}\\ -E\{y_{m}(t)y_{q}^{*}(t)\}E\{y_{n}^{*}(t)y_{p}(t)\},m,n,p,q\in (-u,-1)\cup (1,u) \end{array}$$

Under the above assumptions (A1) and (A2) and by using the properties of cumulants, we have

(9) $$cum(y_{m}(t),y_{n}^{*}(t),y_{p}(t),y_{q}^{*}(t))=\sum_{k=1}^{K}e^{j[(x_{m}-x_{n}+x_{p}-x_{q})\omega_{k}+(x_{m}^{2}-x_{n}^{2}+x_{p}^{2}-x_{q}^{2})\varphi_{k}]}c_{4,sk}$$

where $c_{4,sk}=cum(s_{k}(t),s_{k}^{*}(t),s_{k}(t),s_{k}^{*}(t))$ represents the kurtosis of the kth source signal. Note that the noise term is vanished because the fourth order cumulant of Gaussian noise is zero. Since we are only interested in the DOAs of sources, it is better to remove the $\varphi_{k}$ term and retain the $\omega_{k}$ term in Equation (9).

Let $x_{n}=-x_{m}$ and $x_{p}=-x_{q}$, and substituting them to Equation (9), we obtain

(10) $$cum(y_{m}(t),y_{-m}^{*}(t),y_{-q}(t),y_{q}^{*}(t))=\sum_{k=1}^{K}e^{j2(x_{m}-x_{q})\omega_{k}}c_{4,sk}$$

The right side of Equation (10) behaves like the correlation between the mth and qth sensor output of a virtual far-field array, which is just the difference co-array of the physical array.

### 2.3. The DOA Estimation Method

If a uniform linear array (ULA) is exploited, we can easily construct a matrix $C_{1}$, the (m,q)th entry of which can be given by

(11) $$C_{1}(\tilde{m},\tilde{q})=\{\begin{array}{l} cum(y_{\tilde{m}-u-1}(t),y_{-\tilde{m}+u+1}^{*}(t),y_{-\tilde{q}+u+1}(t),y_{\tilde{q}-u-1}^{*}(t)),\;1\leq \tilde{m},\tilde{q}\leq u\\ cum(y_{\tilde{m}-u-1}(t),y_{-\tilde{m}+u+1}^{*}(t),y_{-\tilde{q}+u}(t),y_{\tilde{q}-u}^{*}(t)),\;1\leq \tilde{m}\leq u,\;u+1\leq \tilde{q}\leq 2u\\ cum(y_{\tilde{m}-u}(t),y_{-\tilde{m}+u}^{*}(t),y_{-\tilde{q}+u+1}(t),y_{\tilde{q}-u-1}^{*}(t)),\;u+1\leq \tilde{m}\leq 2u,1\leq \tilde{q}\leq u\;\\ cum(y_{\tilde{m}-u}(t),y_{-\tilde{m}+u}^{*}(t),y_{-\tilde{q}+u}(t),y_{\tilde{q}-u}^{*}(t)),\;u+1\leq \tilde{m},\tilde{q}\leq 2u \end{array}$$

According to [10], $C_{1}$ can be represented in a compact form as

$$C_{1}=B\Lambda B^{H}$$

where, $b(\omega_{k})={\left[e^{-j2u\omega_{k}},e^{-j2(u-1)\omega_{k}},\cdots ,e^{j2u\omega_{k}}\right]}^{T}$ and $\Lambda =diag\{c_{4,s1},c_{4,s2},\cdots ,c_{4,sK}\}$. Note that the matrix $C_{1}$ behaves like the covariance matrix of the received signals by a far-field array, where the array manifold and steering vector are given by the matrix B and the vector $b(\omega_{k})$. Then, the conventional MUSIC method [2] can be utilized to find the DOAs of the sources. However, with an array of N elements the maximum number of sources that the method can detect is N−1, hence it is not applicable to the case of underdetermined DOA estimation. Note that according to Equation (10), the difference co-array of a physical array is acquired. Therefore, non-ULA can be employed so as to further increase the resolution and the number of sources to be detected.

## 3. The Proposed Array Geometry and Method

### 3.1. Nested Array

In literature [17], a new array geometry called nested array was proposed to locate far-field sources. Through the use of nested array, up to $O(N^{2})$ far-field sources with N sensors can be detected while obtaining relatively high resolution. Nested array [17] is basically a concatenation of two ULAs: inner and outer where the inner ULA has $N_{1}$ elements with spacing d and the outer ULA has $N_{2}$ elements with spacing $\bar{d}$ such that $\bar{d}=(N_{1}+1)d$. More precisely, it is a linear array with sensor location given by $S_{inner}=\{md,m=1,2,\cdots ,N_{1}\}$ and $S_{outer}=\{n(N_{1}+1)d,n=1,2,\cdots ,N_{2}\}$. It has been shown that the difference co-array of nested array is a filled ULA with $2N_{2}(N_{1}+1)-1$ elements [17], which implies that the degrees of freedom (DOF) is $2N_{2}(N_{1}+1)-1$. Unfortunately, we cannot exploit nested array directly in the near field since the derivation of Equation (10) is based on a symmetric array.

### 3.2. Symmetric Nested Array

In order to take advantage of nested array in the near-field, a simple approach is to locate two identical nested arrays symmetrically. For convenience, we call it a symmetric nested array (SNA). The sensor location of a SNA is given by $S_{inner}=\{md_{1},m=-N_{1},-N_{1}+1,\cdots ,-1,1,\cdots ,N_{1}\}$ and $S_{outer}=\{n(N_{1}+1)d_{1},n=-N_{2},-N_{2}+1,\cdots ,-1,1,\cdots ,N_{2}\}$. From the property of nested array, the difference co-array of symmetric nested array with $2(N_{1}+N_{2})$ elements contains a ULA with $2N_{2}(N_{1}+1)-1$ elements, where $N_{1}$ and $N_{2}$ represent the number of sensors of the inner and outer ULA in each nested array, respectively.

### 3.3. Compressed Symmetric Nested Array

This subsection introduces a new array geometry which is based on nested array. Note that a nested array consists of two ULAs, where the inner ULA and the first elements of the outer ULA also constitute a ULA. Since ULA is always symmetric, we construct the new symmetric nested array as follows.

**Figure 2:**
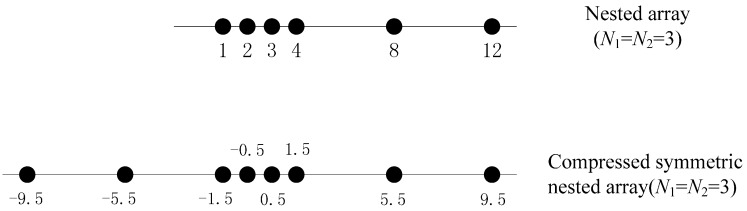
Nested array ($N_{1}=3,N_{2}=3$) and compressed symmetric nested array ($N_{1}=3,N_{2}=3$). The difference co-array of the two arrays contains the same ULA.

First, it is assumed that the inner and outer ULA in a nested array have $N_{1}$ and $N_{2}$ elements, respectively. Since the inner ULA and the first element of the outer ULA also compose of a new ULA with $N_{1}+1$ elements, we take the centroid of the new ULA as the phase reference. Then a ULA with $N_{2}-1$ elements is added to the left of the nested array to make the non-ULA symmetrically. Hence, the final non-ULA has $N=N_{1}+2N_{2}-1$ elements. Compared with a symmetric nested array, fewer sensors are used in the final non-ULA while achieving the same DOF, so we call it a compressed symmetric nested array (CSNA). More specifically, the sensor location of a CSNA is given by the union of the sets $S_{1}=\{(m-\frac{N_{1}+1}{2})d,m=1,\cdots ,N_{1}+1\}$, $S_{2,r}=\{[m(N_{1}+1)-\frac{N_{1}+1}{2}]d,m=2,\cdots ,N_{2}\}$ and $S_{2,l}=\{[m(N_{1}+1)+\frac{N_{1}+1}{2}]d,m=-N_{2},\cdots ,-2\}$. To make the definition clearly, an example of a CSNA with 8 elements is depicted in Figure 2. Apparently a CSNA contains a nested array, so it can achieve the same DOF as the contained nested array.

It has been shown that a nested array can attain $2N_{2}(N_{1}+1)-1$ DOF in the difference co-array using only $N_{1}+N_{2}$ elements [17]. Similarly, we can find a systematic way to increase the DOF of the difference co-array in the near-field. The distribution of sensors can be further optimized by finding $N_{1}$ and $N_{2}$ that maximize the total DOF $2N_{2}(N_{1}+1)-1$, under the constraint of fixed total number of sensors, i.e., $N=N_{1}+2N_{2}-1$. The solution can be verified as seen in Table 1.

### 3.4. DOA Estimation Using CSNA in the Near-Field

As Equation (10) shows, the correlation of the difference co-array of the physical array is acquired. More specifically, if the physical array sensor locations are spatially distributed in such a way that the elements in the set of differences $\left\{x_{m}-x_{q},-u\leq m,q\leq u\right\}$ represent every integer from 0 to $N_{v}$, where $N_{v}\leq N(N-1)/2$ is an integer, then we have $N_{v}+1$ autocorrelation lags given by

(12) $$c(l)=c(x_{m}-x_{q})=\sum_{k=1}^{K}e^{2jl\omega_{k}}c_{4,sk},l=0,\cdots ,N_{v}$$

Interestingly, these autocorrelation lags are identical to those corresponding to a uniform linear array of $N_{v}+1$ elements for the same source scene in the far-field.

When a CSNA with $N=N_{1}+2N_{2}-1$ elements is employed, it can be verified that its difference co-array contains the same ULA with $2N_{2}(N_{1}+1)-1$ elements as that of the corresponding nested array, which implies that $N_{v}=\frac{DOF-1}{2}=N_{2}(N_{1}+1)-1$ sources can be identified using a CSNA with $N=N_{1}+2N_{2}-1$ elements.

Then, we can construct a matrix:

(13) $$C=\left[\begin{array}{cccc} c(0) & c(1) & \cdots & c(N_{v})\\ c^{*}(1) & c(0) & \cdots & c(N_{v}-1)\\ \vdots & \vdots & \ddots & \vdots \\ c^{*}(N_{v}) & c^{*}(N_{v}-1) & \cdots & c(0) \end{array}\right]$$

The matrix C has the same form as the conventional covariance matrix of the output of the ULA with $N_{v}+1$ elements whose steering vector can be represented as $\tilde{a}(\theta_{k})=[1,e^{j2\omega_{k}},\cdots ,e^{j2N_{v}\omega_{k}}]$. Then, the conventional MUSIC method can be used to estimate the DOAs. Let $U_{n}$ denote the noise space spanned by the eigenvectors corresponding to the small eigenvalues. The spatial spectrum can be defined as

(14) $$P(\theta )=\frac{1}{{\tilde{a}}^{H}(\theta )U_{n}U_{n}^{H}{\tilde{a}}^{H}(\theta )}$$

As a result, the DOAs can be obtained by finding the first K peaks of P(θ).

Given the measured data {y(t),t=1,2,⋯,T}, the proposed DOA estimation method using CSNA can be summarized as the following steps.

- Use Equation (8) to compute the fourth order cumulants of the observed signals;
- Find $c(0),c(1),\cdots ,c(N_{v})$ from the cumulants;
- Construct matrix C using Equation (13);
- Compute the spatial spectrum P(θ) using Equation (14);
- Find the first K peaks of spatial spectrum P(θ).

*Remarks:*

1. When SNA is exploited in Equation (12), we can also construct a virtual covariance matrix like Equation (13) and use the conventional MUSIC method to estimate the DOAs. A subspace-based DOA estimation method using a SNA with $N=2(N_{1}+N_{2})$ elements can detect $N_{2}(N_{1}+1)-1$ sources. It is obvious that the method based on SNA can identify more sources than sensors when $N_{1}\geq 3$ and $N_{2}\geq 3$.
2. Since only one half of the difference co-array is employed in our method, the proposed method can detect $N_{2}(N_{1}+1)-1$ sources with $N=N_{1}+2N_{2}-1$ elements. Compared with the SNA, fewer sensors are acquired for CSNA to detect the same number of sources.
3. Regarding the computational complexity of the proposed method, the main cost is in calculating cumulants and eigenvalue decomposition (EVD) of matrix C. Calculation of cumulants and EVD of matrix C requires $O(TN^{4})$ and $O({\left(N_{v}+1\right)}^{3})$, respectively. When different array geometry is utilized, the dimension of matrix C is also different. Without loss of generality, we assume the number of sensors N=4k, where k is an integer. The value of $N_{v}+1$ is presented in Table 2 for ULA, SNA and CSNA respectively.

Therefore, the computational cost of the proposed method based on the CSNA is $O(TN^{4})+O\left({\left[\frac{N^{2}}{8}+\frac{N}{2}\right]}^{3}\right)$, and is somewhat higher than that of the proposed method based on the SNA, where the cost is $O(TN^{4})+O\left({\left[\frac{N^{2}}{16}+\frac{N}{4}\right]}^{3}\right)$. It is also higher than that of the method in [10], where the main cost is $O(TN^{4})+O(N^{3})$. However, the advantages of the proposed method based on the CSNA include high resolution and the ability to detect more sources than sensors.

## 4. Simulation Results

In this section, we provide some numerical experiments to illustrate the superior performance of the proposed method based on CSNA. In the following figures, the ULA, S-nested array and CS-nested array stand for the method in [10], and the proposed method using the SNA and CSNA, respectively. First of all, an example will be given to demonstrate the underdetermined ability of the proposed method using the CSNA. Then we make a comparison of the three methods in terms of computational cost, RMSE and resolution ability. The source signals and noise are modeled as an exponential process and Gaussian white noise, respectively. The intersensor spacing of the ULA is $d=\frac{\lambda }{4}$, which is equivalent to the underlying grid of minimum spacing of the CSNA and SNA.

### 4.1. Underdetermined DOA Estimation

**Figure 3:**
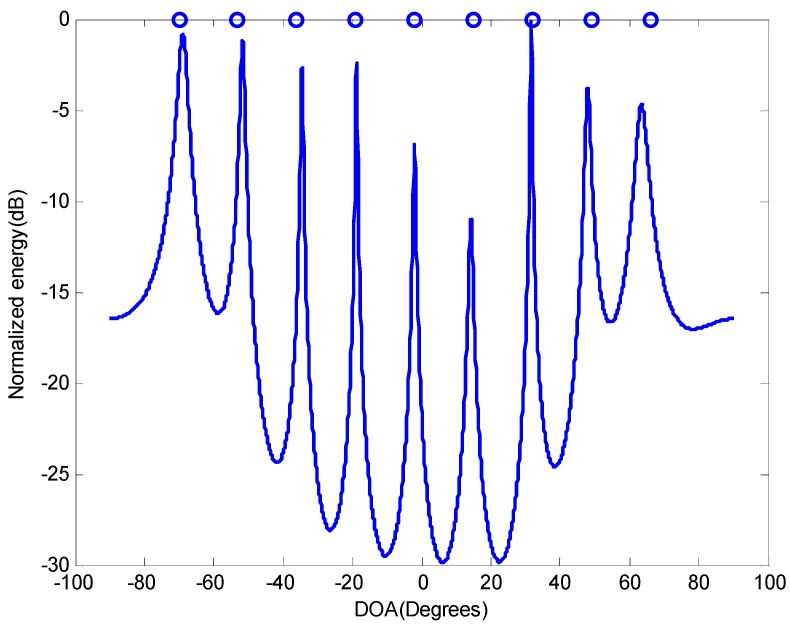
Spatial spectrum for underdetermined DOA estimation, K=9,N=8.

First, consider the scenario in which a CSNA with 8 sensors receives 9 narrowband sources radiated from {(−70°,13λ),(−36°,14λ),(−19°,16λ),(−53°,12λ),(−2°,18λ),(15°,20λ),(32°,19λ),(49°,17λ),(66°,15λ)}. According to Section 3, the DOF of our method based on a CSNA with 8 sensors is increased to 12, thus can estimate DOAs of 11 sources. Figure 3 plots the spatial spectrum of the proposed method based on the CSNA shown in Figure 2 with SNR=20dB and T=4000. It can be seen that the proposed method can resolve the 9 signals clearly with 8 sensors.

### 4.2. Computational Cost

Next, we consider a case in which two uncorrelated sources radiated from {−11°,14λ} and{8°,16λ} impinge upon three different linear array with 8 elements, which are the CSNA shown in Figure 2, the SNA with sensor number in each level $N_{1}=N_{2}=2$ and a ULA. The computational time of the above three methods for finding the DOAs are illustrated in Table 3. By averaging 100 trials, this experiment is implemented in Matlab on windows 7 and a computer with Intel core i5-4590 CPU with 1000 snapshots and a search grid 0.1°. It can be seen that the time consumptions of the three methods are very close in that the main cost of these methods is in calculating cumulants. Besides, it can be also clearly seen that the proposed method based on CSNA requires a little more time than the other two methods, which verifies the theoretical analysis in Section 3. However, by using the CSNA with 8 sensors, the DOF in the proposed method can be increased to $\frac{N^{2}}{4}+N-1=23$ and thus as many as 11 sources, even more than the number of physical sensors, can be resolved. Our proposed method using the SNA can only identify 5 sources because the DOF is only $\frac{{\left(\frac{N}{2}\right)}^{2}-2}{2}+\frac{N}{2}=11$ with 8 physical sensors. Furthermore, the proposed method based CSNA can achieve higher resolution than the other two methods, which will be verified in the Section 4.4.

### 4.3. RMSE versus SNR

**Figure 4:**
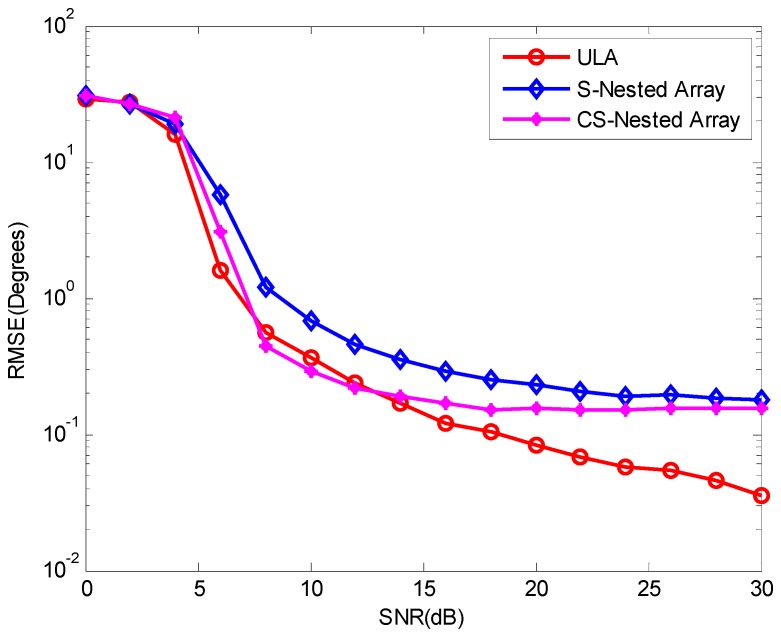
RMSE as a function of SNR for three methods.

Now, let us take a comparison of the three methods by evaluating the RMSE of the DOA estimates as a function of SNR. The RMSE is defined as

$$RMSE=\sqrt{\frac{1}{KN_{mc}}\sum_{q=1}^{K}\sum_{i=1}^{N_{mc}}\left({\hat{\theta }}_{q,i}-\theta_{q,i}\right)}$$

where $N_{mc}$ denotes the number of Monte Carlo trials and ${\hat{\theta }}_{q,i}$ and $\theta_{q,i}$ denote the estimate DOA and the real DOA of the qth signal in the ith trial. Consider two sources located at (−25.4°,21λ) and (14.7°,13λ) impinge on the three linear arrays with 8 elements used in previous experiment. Figure 4 shows the RMSE versus SNR of the three methods by averaging 200 Monte Carlo trials and calculating 2000 snapshots. It can be seen that the three methods has nearly the same variance in low SNR and the method in [10] shows better performance than the other two methods in moderate and high SNR. Besides, it is easy to discover that the proposed method based on CSNA has lower estimation error compared with the method based on SNA in moderate and high SNR.

### 4.4. Resolution Ability versus SNR and the Number of Snapshots

**Figure 5:**
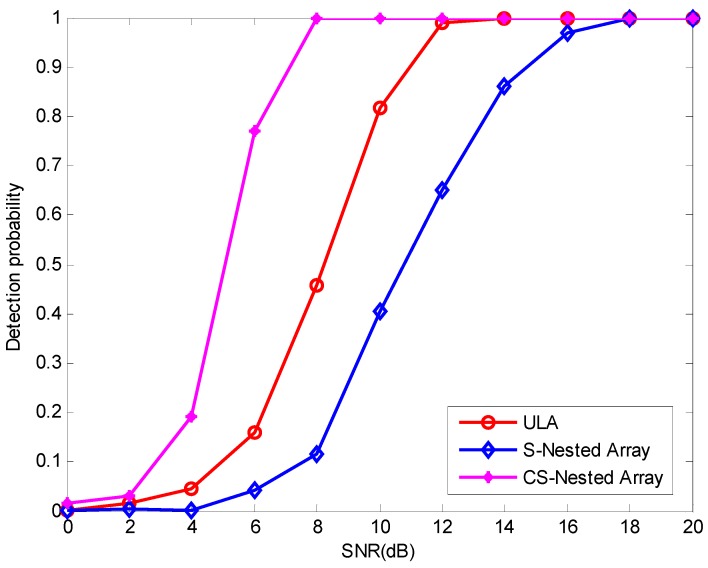
Comparison of resolution ability among the three methods.

To investigate the resolution ability of the proposed method using CSNA, two closely spaced signals with DOAs (−4°,4°) were impinged on each array. The two sources are considered to be resolved in a trial if both $\left|{\hat{\theta }}_{2}-\theta_{2}\right|$ and $\left|{\hat{\theta }}_{1}-\theta_{1}\right|$ are smaller than $\left|\theta_{2}-\theta_{1}\right|/2$, where $\theta_{1}$ and $\theta_{2}$ denote the true DOAs and ${\hat{\theta }}_{1}$ and ${\hat{\theta }}_{2}$ denote the estimating DOAs. The detection probability of the three methods versus SNR is depicted in Figure 5, where the number of snapshots is T=2000 and 600 Monte Carlo trials are carried out. As Figure 5 shows, the method using the CSNA gets a higher resolution ability compared with the other two methods, mainly because the CSNA can achieve higher DOF than the others.

**Figure 6:**
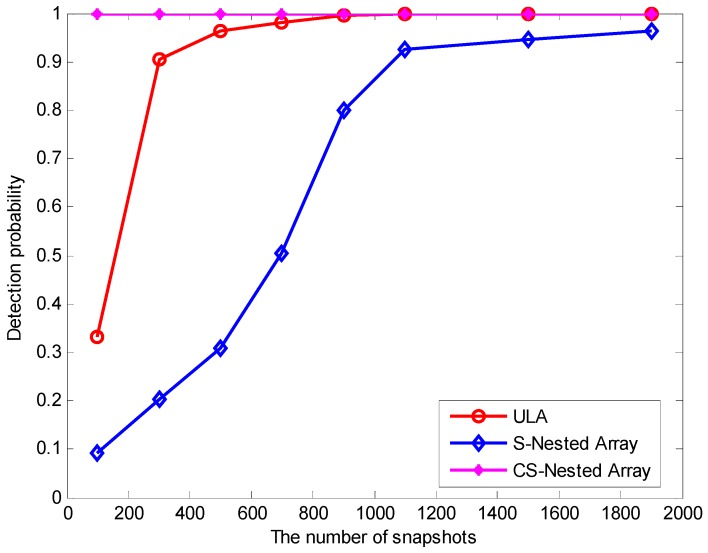
Detection probability versus total number of snapshots for the three methods.

Figure 6 illustrates the detection probability of the three methods as a function of the number of snapshots, where the parameter are kept the same as before except SNR = 25 dB and DOAs=(−3°,3°). From Figure 6, it can be clearly observed that, the proposed method based on CSNA outperforms the two other methods due to higher DOF.

## 5. Conclusions

In this paper, a novel underdetermined DOA estimation method is proposed based on fourth-order cumulants to achieve superior resolution in the near field. A new array geometry, called a compressed symmetric nested array, is employed so that as many as $N_{2}(N_{1}+1)-1$ sources can be detected using only $N=N_{1}+2N_{2}-1$ sensors in the near field. Although our proposed method leads to somewhat higher variance than some other methods due to exploiting parts of the cumulants, it obtains higher resolution. In future work, we will try to utilize all fourth-order cumulants to improve the estimation accuracy.

## Figures and Tables

**Table 1:** The relationship between the number of sensors and DOF of CSNA.

The Number of Sensors (k is an Integer)	The Number of Sensors of Inner ULA	The Number of Sensors of the Outer ULA	The DOF Achieved
N=4k	$N_{1}=\frac{N}{2}+1\;$	$N_{2}=\frac{N}{4}$	$\frac{N^{2}}{4}+N-1$
	$N_{1}=\frac{N}{2}-1$	$N_{2}=\frac{N}{4}+1$	
ss N=4k+1	$N_{1}=\frac{N-1}{2}$	$N_{2}=\frac{N+3}{4}$	$\frac{N^{2}-1}{4}+N$
N=4k+2	$N_{1}=\frac{N}{2}$	$N_{2}=\frac{N+2}{4}$	$\frac{N^{2}}{4}+N$
N=4k+3	$N_{1}=\frac{N+1}{2}$	$N_{2}=\frac{N+1}{4}$	$\frac{N^{2}-1}{4}+N$

**Table 2:** The value of $N_{v}+1$ for ULA, SNA and CSNA.

**Array Geometry**	$N_{v}+1$
ULA	N=4k
SNA	$\frac{N^{2}}{16}+\frac{N}{4}=k^{2}+k$
CSNA	$\frac{N^{2}}{8}+\frac{N}{2}=2k^{2}+2k$

**Table 3:** The time required for estimating the DOAs of three methods.

Methods	Time Consumptions (s)
ULA	0.2719
S-Nested array	0.2687
CS-Nested array	0.2743
